# No

**Published:** 1923-02

**Authors:** E. W. Morris

**Affiliations:** C.B.E., (House Governor, London Hospital)


### NO.—By E. W. MORRIS, C.B.E.

**(House Governor, London Hospital.)**

THE knowledge gained by the experiment of a Combined Appeal is such, in my opinion, as to lead one to oppose any repetition of the experiment. In saying this I wish it to be understood that I am not finding fault with the way this particular combined appeal has been run. If there is to be a combined appeal at all I cannot imagine any other way of running it. I am objecting to a combined appeal of any kind. And for two reasons.

### The Personal Factor.

First, because all gifts to hospitals seem to be of a personal character. The rich give to some particular hospital because for generations members of the family have been Life Governors and Members of the Board. The poor give to some particular hospital because it is theirs, their very own; in it their boy was saved; the mother was snatched from the very brink; the father underwent some serious operation; some dear one, after a gallant fight, passed away. I have never been more impressed than during the year just passed by this strong sentiment. A combined appeal, run from a non-hospital address, will always be handicapped by the absence of this personal feeling. I suppose I am not the only hospital official who has met people in 1922 who would willingly give to one hospital, but strongly objected to their gift being shared with some other hospital in which they had no sort of interest. The number of gifts made to the Combined Appeal which were earmarked to some particular hospital tells the same story, and it may be a surprise to such selective donors to know that the hospital of their choice may not have benefited at all by their kindly action. Were we not told at one of the meetings that a donor was quite at liberty to earmark his £5,000 to, say, "X" Hospital, but that the Distribution Committee, in making its award, would have to remember that "X" Hospital was £5,000 better off, and would make its allotment accordingly?

### Where the Advertising Failed.

My second objection is that non-hospital people cannot make hospital appeals. There is an unwritten and subtle code of etiquette in hospital life, and to ride rough-shod over this is only asking for trouble. I cannot imagine that a hospital man would ever have suggested that a procession of hospital committees and hospital honorary staffs should march about the West End with bands and banners. The thing was a laughing stock. We all had our little joke about it. We were all going to hire substitutes for the day and dress them up in hired academic robes. The lack of all originality in the appeal was another of the effects of working it with non-hospital people. Every hospital has tried practically every "stunt" that was attempted, except perhaps the one of sending wireless messages to incoming liners. Bazaars, garden parties, dances, City dinners, special theatrical shows, sports, football matches, boxing bouts, military tattoos and fireworks, scientific exhibitions, processions, dressed-up students, flag days, cinema pictures of hospital incidents, advertisements on tickets, trick collecting boxes, puzzle competitions, organised collections—we knew them all by heart, their value and their pitfalls. Their day is past. The broadcast advertising showed the same lack of originality, and this was due to the same cause. It has made the public weary of hopsitals. And yet, in spite of this broadcasting, these advertisements never told the real aim and needs of the hospitals. The real needs can only be told, so I think, by the hospitals themselves, and by those who work in them and have some ideals, almost passionate ideals, as to their future development. Advertisements in general terms, which would apply equally well to a Rescue Home, a Boys' Home, or a Dogs' Home, or any other charity, will not do for hospital work.

### No Financial Gain.

Those of us who had occasion to visit the offices of the Appeal could not but have been struck by the noise and clatter, and I think we came away with the impression that that sort of work—three or four telephones in a room and as many organisers dictating at the same time—was not the sort of work which could do the hospitals real good. We really cannot make a business of appeal. The heart-beat must be there. And I think this shows in the results. I did not intend to refer to figures at all, but to speak only of general principles; but I find that I must. I suppose the total result of the Combined Appeal will be somewhere about £450,000. This appears to me a very poor sum considering its object—the hundred hospitals of London—and considering, too, the machinery used to raise it. Every five years my own hospital, "The London," makes a special Quinquennial Appeal. The last was in 1918. The result of that appeal, run entirely by ourselves, was that about £150,000 was received from friends of the hospital in donations and subscriptions. The receipts of the Combined Appeal ought to have been a great deal more than only three times the amount raised by one single hospital. Moreover, this £450,000 is very far from being new money; it is our money, withdrawn from us, and sent to a Central Body, which re-distributes it to us. We were all asked to do no special appealing ourselves, and we loyally agreed. I do not know what other hospitals have found, but we at the London are losers by the arrangement. In 1921, the year before the Combined Appeal, our donations and boxes were about £107,000. In 1922, the year of the great appeal, our own donations and subscriptions fell to £46,000 (up to December 1). We have received less than £16,000 from the Combined Appeal. I admit that 1921 was a good year. Nevertheless, our average in donations for the last 10 years has been £63,623. Obviously the Appeal has done us harm, and I am afraid the financial harm is not the only one. It has spoiled the chances of our own appeals for 1923, because the public are sick to death of the hospitals; and yet I doubt whether the public know any more to-day of the real life history of a hospital, its ideals, its victories, its fears and its tremendous importance to the Nation than it did at the beginning of 1922. The public were not likely to know much from any poster or advertisement I saw. The slogan "Save your Hospitals" irritated, because it begged the question that there was a reason why they should be saved.

### "My" Hospital.

In ten years' time the public may understand that a hospital system is worth saving, and then a hospital general appeal may be successful. At present they do not appear to know it: they do not recognise a hospital system, but only "my" hospital. It seems wise, therefore, to appeal to a sentiment that exists. It is of little use for non-hospital people to appeal to one that is not yet born.

---

A Cottage Hospital Conference.—Mr. C. E. Howe, Chairman, and Mr. F. Nunn, Honorary Secretary of Colwyn Bay Cottage Hospital, convened a conference of representatives of North Wales Hospitals. The meeting was well attended, and upon a set of forms that had been prepared in advance the practice of the different hospitals, large and small, were tabulated. A long list of subjects was discussed. The meetings were held in the Magistrates' Room in the County Buildings; and are interesting as an apparently spontaneous desire for co-ordination, not springing from any society or association for the purpose.

